# Cognitive Processes Underlying Verbal Fluency in Multiple Sclerosis

**DOI:** 10.3389/fneur.2020.629183

**Published:** 2021-01-21

**Authors:** Alfonso Delgado-Álvarez, Jordi A. Matias-Guiu, Cristina Delgado-Alonso, Laura Hernández-Lorenzo, Ana Cortés-Martínez, Lucía Vidorreta, Paloma Montero-Escribano, Vanesa Pytel, Jorge Matias-Guiu

**Affiliations:** Department of Neurology, Instituto de Investigación Sanitaria San Carlos (IdISSC), Hospital Clínico San Carlos, Universidad Complutense, Madrid, Spain

**Keywords:** multiple sclerosis, cognitive, neuropsychology, fluency, processing speed, machine learning

## Abstract

**Background:** Verbal fluency (VF) has been associated with several cognitive functions, but the cognitive processes underlying verbal fluency deficits in Multiple Sclerosis (MS) are controversial. Further knowledge about VF could be useful in clinical practice, because these tasks are brief, applicable, and reliable in MS patients. In this study, we aimed to evaluate the cognitive processes related to VF and to develop machine-learning algorithms to predict those patients with cognitive deficits using only VF-derived scores.

**Methods:** Two hundred participants with MS were enrolled and examined using a comprehensive neuropsychological battery, including semantic and phonemic fluencies. Automatic linear modeling was used to identify the neuropsychological test predictors of VF scores. Furthermore, machine-learning algorithms (support vector machines, random forest) were developed to predict those patients with cognitive deficits using only VF-derived scores.

**Results:** Neuropsychological tests associated with attention-executive functioning, memory, and language were the main predictors of the different fluency scores. However, the importance of memory was greater in semantic fluency and clustering scores, and executive functioning in phonemic fluency and switching. Machine learning algorithms predicted general cognitive impairment and executive dysfunction, with F1-scores over 67–71%.

**Conclusions:** VF was influenced by many other cognitive processes, mainly including attention-executive functioning, episodic memory, and language. Semantic fluency and clustering were more explained by memory function, while phonemic fluency and switching were more related to executive functioning. Our study supports that the multiple cognitive components underlying VF tasks in MS could serve for screening purposes and the detection of executive dysfunction.

## Introduction

Multiple sclerosis (MS) is a demyelinating disease and the most common cause of non-traumatic disability in working-age adults (1). It presents different lesions and cortical/ subcortical gray matter brain damage, as well as functional disconnection (2). The most prominent cognitive symptoms are slowed cognitive processing speed, attention, episodic memory, and executive function impairments, including verbal fluency (VF) deficits, and visuospatial analysis impairment (3).

Executive functions are an essential part of cognitive assessment and include different specialized cognitive processes. One of these cognitive processes is fluency, understood as the ability to generate non-overlearned responses after a cue presentation in a certain time window (4). In this regard, verbal fluency tasks are some of the most widely used tasks, and according to the cue presentation, it is possible to distinguish two modalities: words of a specific semantic field, called semantic fluency; and words beginning with a specific letter, named phonemic fluency. Due to the time window of the task, sustained activation is necessary for the generation of non-overlearned responses (also called processes of energization). While search and access strategies are required during fluency tasks (4), selection mechanisms seem to be key to understanding the different mechanisms involved in phonemic and semantic fluency. Phonemic fluency tasks imply a selection effort to retrieve words according to the initial letter, instead of semantic fields that are more common. Thus, associated stored words could be easily activated and should be inhibited based on task instruction. In contrast, a semantic cue would activate interconnected words based on lexico-semantic networks, giving as a result, less competition between correct words and intrusions than in a phonemic fluency task (5, 6). For this reason, deficits in phonemic fluency tasks have been more closely associated with executive dysfunction. On the other hand, deficits in semantic fluency tasks could be more related to semantic memory impairments than executive dysfunction (7). After word retrieval, self-monitoring processes play a significant role in verbal fluency tasks (4, 7).

Although verbal fluency tasks have been more studied than other fluency tasks, the cognitive processes involved in VF remain unclear (5). In this regard, the limited information of a total score (number of correct answers) has given rise to the study of other scores, such as word production during the first 15 s and errors. The higher number of words during the first 15 s, compared to the decrease in word production during the rest of the time window suggests easy access to the lexico-semantic storage and the need for a search strategy to continue the word production over the course of the task (8). Errors have also been proposed as complementary information in VF. Repetitions and intrusions (also called rule break errors) are the most frequent and have been associated with inhibition impairments (8). However, these scores do not give specific information about the lexical access strategy (9) and there is not a significant difference in error score between MS patients and healthy controls to consider errors as an optimal executive dysfunction measure (8). For a deeper understanding of the lexical access strategy, it has been proposed the study of clustering and switching (7). During task performance, participants generate different responses that can be classified into subcategories or clusters. Once a subcategory is exhausted, participants switch to a different subcategory (7). Thus, it is possible to obtain the number of clusters and switches, as well as qualitative information. These parameters could be more sensitive to detect the underlying neuropsychological deficits involved in each patient and could contribute to the understanding of cognitive function in patients with MS (10).

The cognitive profile of MS is generally characterized by impairments in processing speed, attention-executive functioning, and memory. VF tasks have some important advantages in clinical practice. They are easy to administer and shorter than other neuropsychological assessments for MS. Because VF has been associated with executive functioning and memory and they are assessed in a limited time, VF could serve as sensitive and brief cognitive function measures in MS, with special interest during early onset of the disease (11). Furthermore, these tasks are well-tolerated and are not significantly impacted by visual or motor impairments (4, 6). However, previous studies show inconsistent evidence (6). On the one hand, some authors have suggested VF as a screening test (9, 12, 13). On the other hand, while some studies suggested an equal impairment between phonemic and semantic fluency (6, 14), others have found a greater impairment in phonemic or semantic fluency (6, 15).

Our hypothesis is that VF reflects multiple cognitive components, and the assessment of different VF tasks and several parameters (number of words, clustering, switching, etc.) could be useful to disentail the cognitive demands underlying each task and score. A comprehensive assessment of VF tasks could be useful to detect patients with cognitive impairment in MS, and the impairment of specific cognitive domains, particularly executive functioning. Accordingly, our aim was 2-fold: first, to evaluate the cognitive processes related to verbal fluency in patients with MS through the identification of predictor variables of verbal fluency scores in a comprehensive neuropsychological battery; second, to develop machine-learning algorithms to predict those patients with cognitive deficits using only VF-derived scores.

## Materials and Methods

### Participants

Two hundred participants with multiple sclerosis (MS) were enrolled in this study, including 146 patients with relapsing-remitting MS (RR), 19 patients with primary progressive MS (PP), and 35 patients with secondary progressive MS (SP). Main demographic and clinical characteristics are shown in Table 1. All participants met the McDonald 2017 criteria (16).

**Table 1:** Demographic and clinical characteristics.

	**MS (*n* = 200)**	**RR (*n* = 146)**	**PP (*n* = 19)**	**SP (*n* = 35)**
Age, years	47.12 ± 9.79	44.85 ± 8.74	54.84 ± 10.44	52.28 ± 9.66
Female, %	70.1%	73.3%	42.1%	72.2%
Education, years	15.28 ± 3.76	15.58 ± 3.57	14.47 ± 3.90	14.5 ± 4.36
EDSS	3.07 ± 1.98	2.26 ± 1.43	4.76 ± 1.46	5.44 ± 1.65
Beck depression invent	13.5 ± 9.34	13.06 ± 9.78	11.89 ± 8.23	14.75 ± 7.98
Fatigue severity scale	44.70 ± 14.95	43.34 ± 15.43	44.57 ± 14.04	50.25 ± 12.26
Year of first relapse	2,001 ± 7.12	2,002 ± 6.88	2,003 ± 6.26	1,996 ± 6.82

*Descriptive data are shown as mean ± standard deviation. MS, multiple sclerosis patients; RR, relapsing-remitting MS patients; PP, primary-progressive MS patients; SP, secondary-progressive MS patients*.

### Neuropsychological Assessment

All participants were evaluated using the neuropsychological battery Neuronorma (17, 18), previously validated for MS in our setting. This battery included the following tests: Digit Span forward and backward, Corsi's Test forward and backward, Trail Making Test A and B (TMT), Symbol Digit Modalities Test (SDMT), Boston Naming Test (BNT), Rey-Osterrieth Complex Figure (ROCF) (copy, free recall after 3 and 30 min delay, and a recognition task), Judgement Line Orientation test (JLO), Stroop Color-Word Interference Test (A: word, B: color, C: interference), Free and Cued Selective Reminding Test (FCSRT) (trial 1 free recall, total free recall, total recall (free recall + cued recall) delayed recall, delayed total recall), Tower of London-Drexel test (ToL) (total moves score, total correct score, total initiation time score, total execution time score, total problem-solving time score), a semantic fluency task (SF) (animals), and a phonemic fluency tasks (PF) (words beginning with “p”). According to this battery, patients were classified as cognitively impaired or cognitively preserved using the previously validated criteria (17). In brief, these criteria define cognitive impairment when at least two cognitive domains are −1.67 standard deviations below the mean, according to age-, sex-, and education-adjusted scores. Similarly, cognitive domains were considered impaired according to the same criteria (17) (see Supplementary Material 1).

Furthermore, Paced Auditory Serial Addition Test (PASAT) and two extra phonemic fluency tasks (words beginning with “m” and “r”) were also performed. In the VF tasks, participants were asked to produce as many words as possible in 1 min, according to the specified cues. One point was assigned for each correct word based on the guidelines by Ledoux et al. (19). In addition, Beck's Depression Inventory (20), and Fatigue Severity Scale (21) were administered.

### Procedure

Patients were evaluated on a single session lasting ~120 min. First, digit span, Corsi's test, and VF tasks were performed and took ~10 min. Next, FCSRT was administrated and, to avoid the interference of other verbal stimuli during the delay, tests without a high verbal load were performed, such as SDMT, TMT, ROCF copy, Stroop, ROCF recall after 3 min, and ToL. FCSRT took ~15 min with a delay of 30 min. The SDMT and Stroop were considered timed tests with time of performance of 90 and 45 s per each Stroop part, respectively. TMT, ROCF copy, and recall after 3 min took ~7 min (for mean time details, see Table 2), while Tower of London test took ~20 min. After the delayed recall of FCSRT and during ROCF 30 min delay, tests with verbal responses were administrated, such as PASAT and BNT with a mean duration of 8 and 15 min, respectively. Then, ROCF recall after 30 min, ROCF recognition task, and JLO were administered. Both ROCF tasks took ~7 min, and JLO had a mean time of administration of 15 min. Finally, patients completed the Beck's Depression Inventory and the Fatigue Severity Scale.

**Table 2:** Main neuropsychological results by the three sub groups.

**Test**	**RR**		**PP**		**SP**	
	**Mean**	**SD**	**Mean**	**SD**	**Mean**	**SD**
Span verbal (F)	6.17	1.27	5.69	1.19	6.11	1.19
Span verbal (B)	4.33	1.03	3.94	.92	4.16	1.25
Corsi's test (F)	5.72	1.00	5.25	1.07	5.53	0.964
Corsi's test (B)	4.99	1.12	4.53	1.02	4.74	0.933
TMT-A	44.58	22.54	70.82	52.83	69.61	48.12
TMT-B	94.15	50.79	131.16	83.09	206.72	236.64
SDMT	40.52	13.33	28.64	12.61	27.00	14.61
BNT	52.24	4.96	49.56	7.40	50.95	5.88
JLO	21.45	4.38	20.25	5.55	18.59	6.35
FCSRT-1FR	9.79	2.23	8.17	2.72	8.21	2.32
FCSRT-TFR	32.06	6.57	26.03	9.13	28.05	8.45
FCSRT-TR	44.30	4.93	39.94	8.90	40.32	6.96
FCSRT-FDR	11.17	3.10	8.28	3.73	8.79	4.14
FCSRT-TDR	14.76	2.01	12.56	3.91	13.21	3.29
ROCF-copy	33.32	5.31	31.51	5.17	29.63	7.95
ROCF-time	147.27	67.42	183.31	93.88	218.84	185.98
ROCF-3 min	15.98	6.83	13.28	6.69	13.89	6.88
ROCF-30 min	15.80	6.60	12.67	6.80	12.92	7.50
ROCF-recog.	19.34	2.10	19.45	1.95	19.37	1.97
Semantic fluen.	21.67	5.69	17.75	6.14	20.73	8.60
P fluency	15.33	5.36	14.47	6.24	15.05	6.83
M fluency	13.11	4.92	11.97	5.41	13.68	6.27
R fluency	13.00	4.79	11.77	4.47	14.38	6.74
Stroop-A	100.66	19.36	82.34	23.99	90.18	27.74
Stroop-B	66.24	13.22	54.03	14.85	61.71	20.78
Stroop-C	39.36	11.76	30.23	11.28	33.00	15.92
ToL-CM	4.56	2.16	3.97	2.62	3.33	2.84
ToL-TM	27.01	20.13	26.03	21.85	32.21	19.78
ToL-IT	79.74	49.28	90.59	69.11	69.64	34.40
ToL-ET	260.28	122.16	348.86	363.31	296.29	121.80
ToL-RT	337.11	127.24	365.86	170.64	365.93	141.03
PASAT-C	44.06	10.55	37.78	11.75	46.38	7.63

*F, forward; B, backward; FCSRT-1FR, Free and Cued Selective Reminding Test (FCSRT) first recall; FCSRT-TFR, FCSRT total free recall; FCSRT-TR, FCSRT total recall; FCSRT-FDR, FCSRT free delayed recall; FCSRT-TDR, FCSRT total delayed recall; ROCF-recog, ROCF recognition task; Semantic fluen, semantic fluency (animals); ToL-CM, Tower of London (ToL) number of correct moves; ToL-TM, ToL number of total moves; ToL-IT, ToL initiation time; ToL-ET, ToL execution time; ToL-RT, ToL resolution time; PASAT-C, number of correct answers*.

All scores obtained from fluency tasks were calculated by two of the authors working independently, and final scores were reached by consensus, according to the scoring criteria developed by Ledoux et al. (19). VF-derived scores included: (a) number of correct answers without repetitions or intrusions; (b) repetitions; (c) intrusions; (d) number of clusters; (e) number of switches; (f) mean clusters (total words in clusters/ number of clusters); (g) percentage of correct words in clusters; (h) correct words in clusters. In PF, the results related to words beginning with “p” were considered singly, as well as in the sum of the results from PF considering the three initial-letters (“p,” “m,” and “r”), as previous studies (22).

### Statistical Analysis

Statistical analysis was performed using SPSS Statistics 22.0. Descriptive data are shown as mean ± standard deviation. Pearson's correlation coefficient (*r*) was used for the analysis of the correlation between quantitative variables. The Pearson *r* coefficient was classified as very low (0–0.29), low (0.3–0.49), moderate (0.5–0.69), high (0.7–0.89), and very high (0.9–1). R software (ggplot) was used to create a heatmap of the correlation matrix. One-way ANOVA and Tukey *post hoc* test were calculated for intergroup differences, considering statistically significant a *p* < 0.05. Automatic linear modeling (LINEAR) procedure was used to identify the neuropsychological tests predictors of VF scores (23). A different model was estimated for each VF score, introducing all Neuronorma tests, PASAT, and phonemic fluency scores as predictor variables. Only variables with *p* < 0.05 were considered predictors.

### Machine Learning Analysis

Two supervised classification algorithms, Support Vector Machine (SVM) with linear kernel and Random Forest (RF) were implemented with Scikit-learn v.0.22.1 in Python v.3.6.9. Six different binary classification tasks were performed depending on the class to predict: the presence of cognitive impairment or cognitive dysfunction in five different cognitive domains (attention and executive functioning, information processing speed, memory, visuospatial function, and language), according to the criteria explained above. Before performing classification, high and very high correlated features -those with a Pearson's coefficient >0.7- were excluded. For each classification task, the dataset was randomly split into training (*n* = 140, 70%) and test (*n* = 60, 30%) sets. The split was made taking into account the distribution of each class. Best hyperparameters of each model were determined carrying out a 5-Fold Cross-Validation Grid Search on the training set. Each best model was then evaluated on its corresponding test set. Models' performance was evaluated in terms of precision, recall, and F1-score values.

### Ethical Approval

The study was conducted with the approval of our hospital's Ethics Committee, and all participants gave written informed consent.

## Results

### VF Across Groups and Correlation With Non-cognitive Characteristics

Considering the classification of MS patients, there was only a significant difference between groups in semantic fluency total scores (*F*_2_ = 5.39; *p* = 0.005). Tukey *post hoc* test showed differences between RR and SP groups with lower scores in SP (*p* = 0.004).

Semantic fluency total score correlated with EDSS score (*r* = −0.284; *p* < 0.001). Phonemic fluency (“p” and “pmr” total scores) also correlated with EDSS (*r* = −0.208; *p* = 0.003 and *r* = −0.191; *p* = 0.008, respectively). There was a significant correlation between semantic fluency total score and depression (*r* = −0.195; *p* = 0.006). Phonemic fluency with “p” total score (*r* = −0.176; *p* = 0.012) and phonemic fluency with “pmr” total score (*r* = −0.210; *p* = 0.003) also correlated with depression.

### Correlation Between VF and Other Neuropsychological Tests

**Figure 1:**
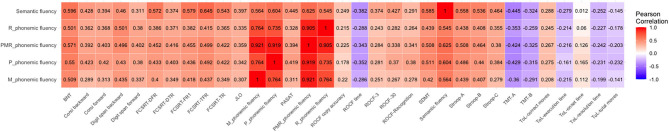
Heatmap of Pearson correlations between fluency tasks and the other neuropsychological tests.

Main neuropsychological results by the three sub groups are shown in Table 2. Correlations between VF and neuropsychological tests are shown in Figure 1. In summary, semantic fluency showed moderate correlations with BNT, FCSRT, phonemic fluency scores, SDMT, Stroop A, and Stroop B. Phonemic fluency with “p” correlated moderately with BNT, SDMT, and semantic fluency. Similar correlations were found in phonemic fluency with “pmr,” including a moderate correlation with Stroop A.

### Neuropsychological Predictors of VF Tests

Automatic linear modeling assessing the neuropsychological predictors of each verbal fluency score is shown in Tables 3–5. The criterion variables with the highest percentage of explanation by the predictor variables were correct answers, clusters, switches, and words in clusters.

**Table 3:** Automatic linear modeling assessing the neuropsychological predictors of semantic fluency.

**Test**	***R^**2**^***	**Variables (transformed)**	**Beta coefficient**	**SE**	***t***	**95% CI**	***P***	**Importance**
Correct answers	0.546	Intercept	−16.895	3.257	−5.18	−23.3, −10.4	<0.001	–
		FCSRT-TFR	0.273	0.058	5.45	0.17, 0.37	<0.001	0.412
		BNT	0.348	0.072	4.84	0.20, 0.49	<0.001	0.324
		Stroop A	0.057	0.017	3.43	0.02, 0.09	0.001	0.164
		PASAT	0.077	0.037	2.08	0.004, 0.14	0.038	0.060
Repetitions	0.117	ToL-TM	0.017	0.004	3.86	0.008, 0.026	<0.001	0.467
		Stroop B	0.020	0.006	3.59	0.009, 0.032	<0.001	0.398
Intrusions	0.05	JLO	0.008	0.003	2.52	0.002, 0.015	0.012	0.443
		BNT	−0.006	0.003	−2.19	−0.012, −0.001	0.029	0.334
Clusters	0.223	Stroop A	0.015	0.006	2.31	0.002, 0.02	0.022	0.286
		Corsi (F)	0.250	0.118	2.11	0.01, 0.48	0.036	0.239
		FCSRT-TFR	0.032	0.016	2.00	0.001, 0.06	0.046	0.216
Switches	0.199	Stroop A	0.030	0.010	2.94	0.01, 0.05	0.004	0.321
		ToL-TM	0.031	0.012	2.55	0.007,0.055	0.011	0.235
		PASAT	0.061	0.024	2.52	0.01, 0.10	0.013	0.229
Mean clusters	0.138	BNT	0.077	0.027	2.88	0.02, 0.13	0.004	0.214
		FCSRT-DFR	0.168	0.060	2.82	0.05, 0.28	0.005	0.204
		TMT-A	0.017	0.006	2.76	0.005, 0.02	0.006	0.195
		Rey-copy	0.093	0.037	2.50	0.02, 0.16	0.013	0.160
		Corsi (F)	−0.319	0.128	−2.49	−0.57, −0.06	0.014	0.159
%words in clusters	0.078	Intercept	46.96	9.827	4.77	27.58, 66.34	<0.001	–
		FCSRT-TR	0.650	0.190	3.41	0.27, 1.02	0.001	0.436
		ToL-PST	0.017	0.007	2.38	0.003, 0.03	0.018	0.213
		ToL-TC	0.879	0.370	2.37	0.15, 1.60	0.018	0.212
Words in clusters	0.463	Intercept	−17.034	3.636	−4.68	−24.20, −9.86	<0.001	–
		FCSRT-TFR	0.268	0.057	4.73	0.15, 0.37	<0.001	0.349
		Stroop B	0.153	0.036	4.24	0.08, 0.22	<0.001	0.280
		BNT	0.329	0.082	4.00	0.16, 0.49	<0.001	0.249

*First column shows criterion variables and the third column shows predictor variables. SE, Standard Error; FCSRT-TFR, Free and Cued Selective Reminding Test (FCSRT)–total free recall; BNT, Boston Naming Test; ToL-TM, Tower of London (ToL)–total moves; JLO, judgment line orientation; Corsi (F), Corsi's test forward; FCSRT-DFR, FCSRT–delayed free recall; TMT-A, Trail Making Test part A; Rey-Copy, Rey-Osterrieth Complex Figure–accuracy on copy; FCSRT-TC, FCSRT–total recall; ToL-PST, ToL–problem-solving time; ToL-TC, ToL–total correct moves*.

**Table 4:** Automatic linear modeling assessing the neuropsychological predictors of phonemic fluency (p).

**Test**	***R^**2**^***	**Variables (transformed)**	**Beta coefficient**	**SE**	***t***	**95% CI**	***P***	**Importance**
Correct answers	0.445	Intercept	−23.43	3.879	−6.04	−31.08, −15.78	<0.001	–
		BNT	0.276	0.074	3.72	0.13, 0.42	<0.001	0.233
		ToL-IT	0.026	0.007	3.52	0.01, 0.04	0.001	0.209
		Stroop A	0.072	0.021	3.44	0.03, 0.11	0.001	0.201
		Corsi (F)	1.037	0.388	2.67	0.27, 1.80	0.008	0.121
		FCSRT-TFR	0.106	0.051	2.09	0.006, 0.20	0.038	0.074
		Rey-Recog	0.334	0.168	1.99	0.004, 0.665	0.048	0.067
Repetitions	0.041	Intercept	0.976	0.324	3.01	0.33, 1.61	0.003	–
		Stroop B	0.009	0.004	2.20	0.001, 0.01	0.029	0.431
		Corsi (B)	−0.125	0.063	−1.99	−0.24, −0.002	0.047	0.353
Intrusions	0.089	Rey-time	0.001	0.000	2.73	0.00, 0.001	0.007	0.285
		Rey-copy	0.012	0.005	2.25	0.001, 0.02	0.025	0.194
		ToL-ET	−0.000	0.000	−2.21	−0.001, 0.00	0.028	0.187
		Span (F)	−0.038	0.018	−2.12	−0.072, −0.003	0.034	0.172
		JLO	−0.008	0.004	−2.06	-0.017, 0.00	0.040	0.162
Clusters	0.204	ToL-ET	−0.003	0.001	−2.95	−0.005, −0.001	0.004	0.365
		FCSRT-TFR	0.038	0.015	2.49	0.008, 0.069	0.013	0.261
Switches	0.307	Intercept	−17.519	3.54	−4.93	−24.51, −10.52	<0.001	–
		BNT	0.210	0.056	3.75	0.10, 0.32	<0.001	0.216
		Stroop A	0.051	0.015	3.43	0.02, 0.08	0.001	0.180
		Corsi (F)	0.833	0.288	2.89	0.26, 1.40	0.004	0.129
		Tol-IT	0.026	0.009	2.87	0.008, 0.04	0.005	0.126
		ToL-ET	0.018	0.007	2.66	0.005, 0.03	0.008	0.109
		FCSRT-1FR	0.292	0.119	2.44	0.05, 0.52	0.015	1.092
		ToL-PST	−0.016	0.007	−2.29	−0.03, −0.002	0.023	0.081
		TMT-B	0.014	0.007	2.10	0.001, 0.02	0.036	0.068
Mean clusters	0.097	Intercept	4.106	0.586	7.00	2.94, 5.26	<0.001	–
		Rey-time	0.005	0.002	3.19	0.002, 0.009	0.002	0.350
		TMT-B	−0.007	0.003	−2.39	−0.013, −0.001	0.018	0.197
		Stroop C	−0.021	0.009	−2.21	−0.039, −0.002	0.028	0.168
		TMT-A	−0.012	0.006	−2.14	−0.024, −0.001	0.037	0.152
% words in clusters	0.136	Intercept	145.94	21.73	6.71	103.08, 188.81	<0.001	–
		Stroop C	−0.59	0.165	−3.58	−0.91, −0.26	<0.001	0.356
		TMT-A	−0.243	0.093	−2.63	−0.42, −0.06	0.009	0.191
		Rey-copy	−1.397	0.547	−2.55	−2.47, −0.31	0.011	0.181
Words in clusters	0.300	ToL-IT	0.040	0.009	4.59	0.02, 0.05	<0.001	0.239
		ToL-PST	−0.014	0.003	−4.54	−0.02, −0.008	<0.001	0.234
		Stroop C	−0.148	0.042	−3.51	−0.23, −0.06	0.001	0.140
		BNT	0.232	0.071	3.25	0.09, 0.37	0.001	0.120
		TMT-A	−0.055	0.018	−3.02	−0.09, −0.01	0.003	0.103
		Span (F)	0.730	0.279	2.61	0.17, 1.28	0.010	0.077
		Stroop B	0.080	0.034	2.35	0.01, 0.14	0.020	0.062

*First column shows criterion variables and the third column shows predictor variables. SE, Standard Error; BNT, Boston Naming Test; ToL-IT, Tower of London (ToL)–initiation time; Corsi (F), Corsi's test forward; FCSRT-TFR, Free and Cued Selective Reminding Test (FCSRT)–total free recall; Rey-Recognition, Rey-Osterrieth Complex Figure (ROCF)–recognition; Corsi (B), Corsi's test backward; Rey-time, ROCF–time on copy; Rey-copy, ROCF–accuracy on copy; ToL-ET, ToL–execution time; Span (F), verbal span forward; JLO, judgement line orientation; FCSRT-1FR, FCSRT–trial 1 free recall; ToL-PST, ToL–problem-solving time; TMT-B, Trail Making Test part B; TMT-A, Trail Making Test part A*.

**Table 5:** Automatic linear modeling assessing the neuropsychological predictors of phonemic fluency (p, r, m).

**Test**	***R^**2**^***	**Variables (transformed)**	**Beta coefficient**	**SE**	***t***	**95% CI**	***P***	**Importance**
Correct answers	0.479	Intercept	−46.12	9.18	−5.02	-64.23, -28.01	<0.001	–
		BNT	0.868	0.179	4.85	0.51, 1.22	<0.001	0.271
		Stroop A	0.215	0.052	4.1	0.11, 0.31	<0.001	0.198
		ToL-IT	0.099	0.028	3.56	0.04, 0.15	<0.001	0.146
		Span (B)	2.85	0.898	3.17	1.07, 4.62	0.002	0.116
		Stroop C	−0.273	0.098	−2.77	-0.46, -0.07	0.06	0.088
		FCSRT-1FR	0.856	0.369	2.31	0.12, 1.58	0.002	0.062
		ToL-PST	−0.045	0.021	−2.12	-0.08, -0.003	0.035	0.052
Repetitions	0.088	FCSRT-TR	−0.048	0.017	−2.92	−0.08, −0.01	0.004	0.361
		ToL-ET	0.006	0.002	2.65	0.002, 0.01	0.009	0.297
Intrusions	0.073	Intercept	1.73	0.382	4.54	0.98, 2.49	<0.001	–
		ToL-ET	−0.002	0.000	−3.51	-0.002, -0.001	0.001	0.550
Clusters	0.293	ToL-ET	−0.007	0.002	−3.51	−0.01, −0.003	0.001	0.240
		Stroop C	−0.082	0.027	−2.98	−0.13, −0.02	0.003	0.173
		Span (B)	0.703	0.253	2.77	0.20, 1.20	0.006	0.149
		Stroop A	0.039	0.014	2.70	0.01, 0.06	0.007	0.142
		BNT	0.124	0.048	2.58	0.02, 0.21	0.010	0.130
		Corsi (F)	0.570	0.273	2.08	0.03, 1.10	0.038	0.085
		ToL-IT	0.011	0.005	2.04	0.00, 0.02	0.042	0.081
Switches	0.328	Intercept	−31.07	6.47	−4.79	−43.84, −18.29	<0.001	–
		BNT	0.58	0.117	4.95	0.34, 0.81	<0.001	0.464
		Stroop A	0.12	0.029	4.44	0.07, 0.18	<0.001	0.374
		Corsi (F)	1.51	0.620	2.44	0.29, 2.74	0.015	0.113
Mean clusters	0.082	Intercept	8.46	2.04	4.15	4.44, 12.49	<0.001	–
		FCSRT-TR	0.108	0.038	2.82	0.03, 0.18	0.005	0.335
		PASAT	0.061	0.027	2.29	0.009, 0.11	0.023	0.222
		TMT-B	−0.014	0.006	−2.18	−0.02, −0.001	0.030	0.201
%words in clusters	0.182	Intercept	193.26	24.51	7.88	144.90, 241.62	<0.001	–
		Stroop C	−1.81	0.350	−5.19	−2.51, −1.12	<0.001	0.513
		Span (B)	11.68	4.03	2.89	3.73, 19.64	0.004	0.160
		ToL-ET	−0.109	0.039	−2.81	−0.18, −0.03	0.005	0.151
		Rey-3 min	1.51	0.595	2.54	0.34, 2.68	0.012	0.123
Words in clusters	0.332	Stroop C	−0.430	0.107	−4.03	−0.64, −0.22	<0.001	0.273
		ToL-ET	−0.026	0.008	−3.28	−0.04, −0.01	0.001	0.181
		ToL-IT	0.054	0.020	2.72	0.01, 0.09	0.007	0.124
		BNT	0.503	0.198	2.54	0.11, 0.89	0.012	0.108
		Stroop B	0.216	0.089	2.43	0.04, 0.39	0.016	0.099
		Span (B)	2.402	1.08	2.21	0.25, 4.54	0.028	0.082

*First column shows criterion variables and the third column shows predictor variables. SE, Standard Error; BNT, Boston Naming Test; ToL-IT, Tower of London (ToL)–initiation time; Span (B), verbal span backward; FCSRT-1FR, Free and Cued Selective Reminding Test (FCSRT)–trial 1 free recall; ToL-PST, ToL–problem-solving time; FCSRT-TR, FCSRT–total recall; ToL-ET, ToL–execution time; Corsi (F), Corsi's test forward; TMT-B, Trail Making Test part B; Rey-3 min, Rey-Osterrieth Complex Figure (ROCF) free recall after 3 min*.

In semantic fluency, the linear modeling identified FCSRT (total free recall), BNT, Stroop A, and PASAT as predictors of correct answers and explained 54.6% or the variance. Regarding cluster score, the model included Stroop A, Corsi's test, and FCSRT (total free recall) and explained 22.3% of the variance. For switches score, the model identified Stroop A, ToL (total moves), and PASAT as predictors, explaining 19.9% of the variance. For words in clusters, FCSRT (total free recall), Stroop B, and BNT was identified as predictors and explained 46.3% of the variance.

In phonemic fluency with “p,” the linear modeling identified BNT, ToL (initiation time), Stroop A, Corsi's test, FCSRT (total free recall), and FCRO (recognition) as predictors of correct answers and explained 44.5% of the variance. For cluster score, ToL (execution time) and FCSRT (total free recall) were identified as predictors, explaining 20.4% of the variance. For switches score, BNT, Stroop A, Corsi's test, ToL (initiation, execution, and problem-solving time), FCSRT (first trial), and TMT-B were identified by the model as predictors and explained 30.7% of the variance. For words in clusters, the model included ToL (initiation, problem-solving time), Stroop C, BNT, TMT-A, verbal span, and Stroop B, explaining 30% of the variance.

In phonemic fluency with “pmr,” BNT, Stroop A and C, ToL (initiation time), verbal span, FCSRT (first trial), and ToL (problem-solving time) were included and explained 47.9% of the variance. For clusters score, the model identified ToL (initiation and execution time), Stroop A and C, verbal span, BNT, and Corsi's test and explained 29.3% of the variance. For switches, BNT, Stroop A, and Corsi's test were included and explained 32.8% of the variance. Finally, the model identified Stroop B and C, ToL (initiation and execution time), BNT, and verbal span as predictors, explaining 32.2% of the variance.

### Machine Learning Classification

**Figure 2:**
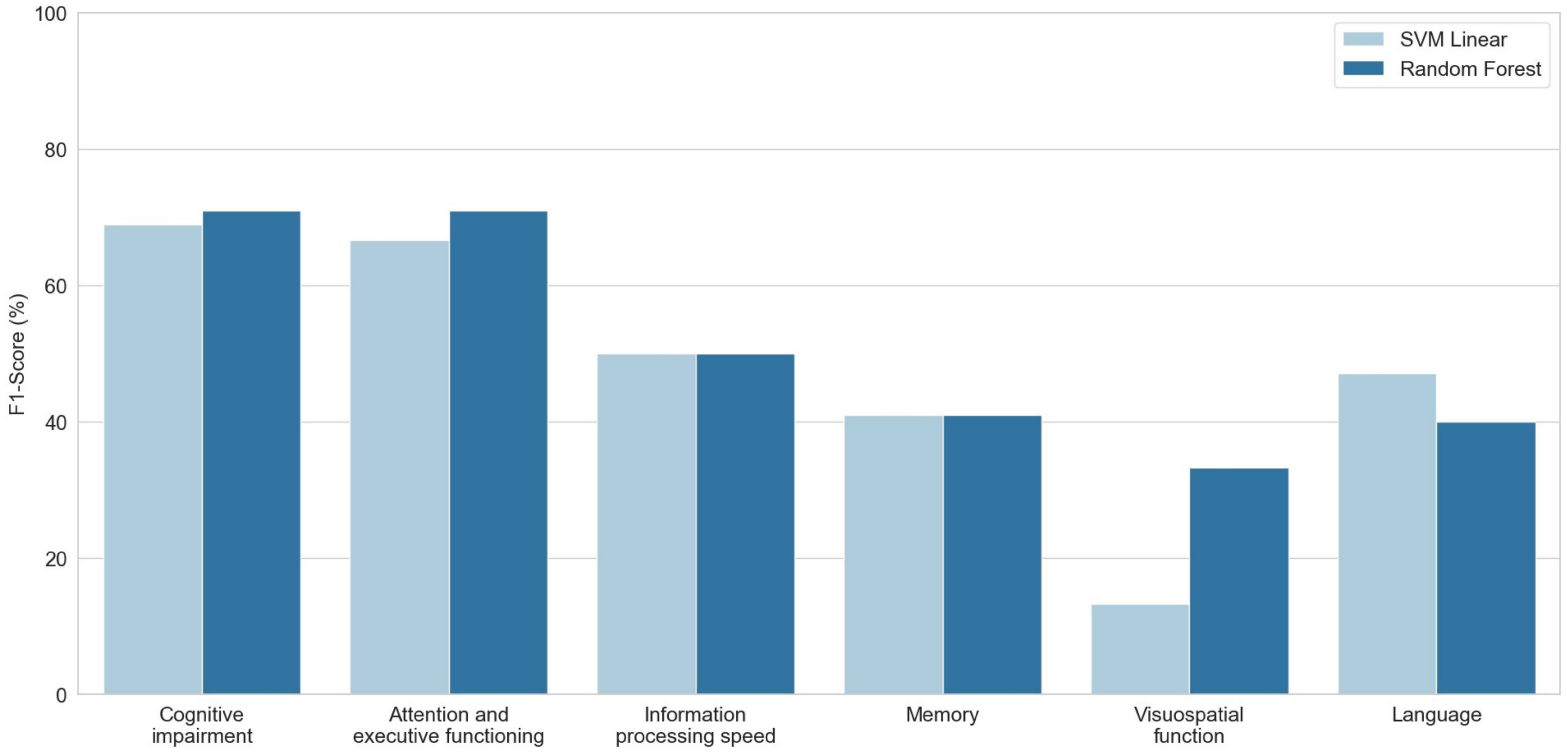
F1-Scores (y-axis) obtained for each classification task (x-axis) using Support Vector Machine with linear kernel (SVM Linear, light blue) and Random Forest (blue) algorithms.

**Figure 3:**
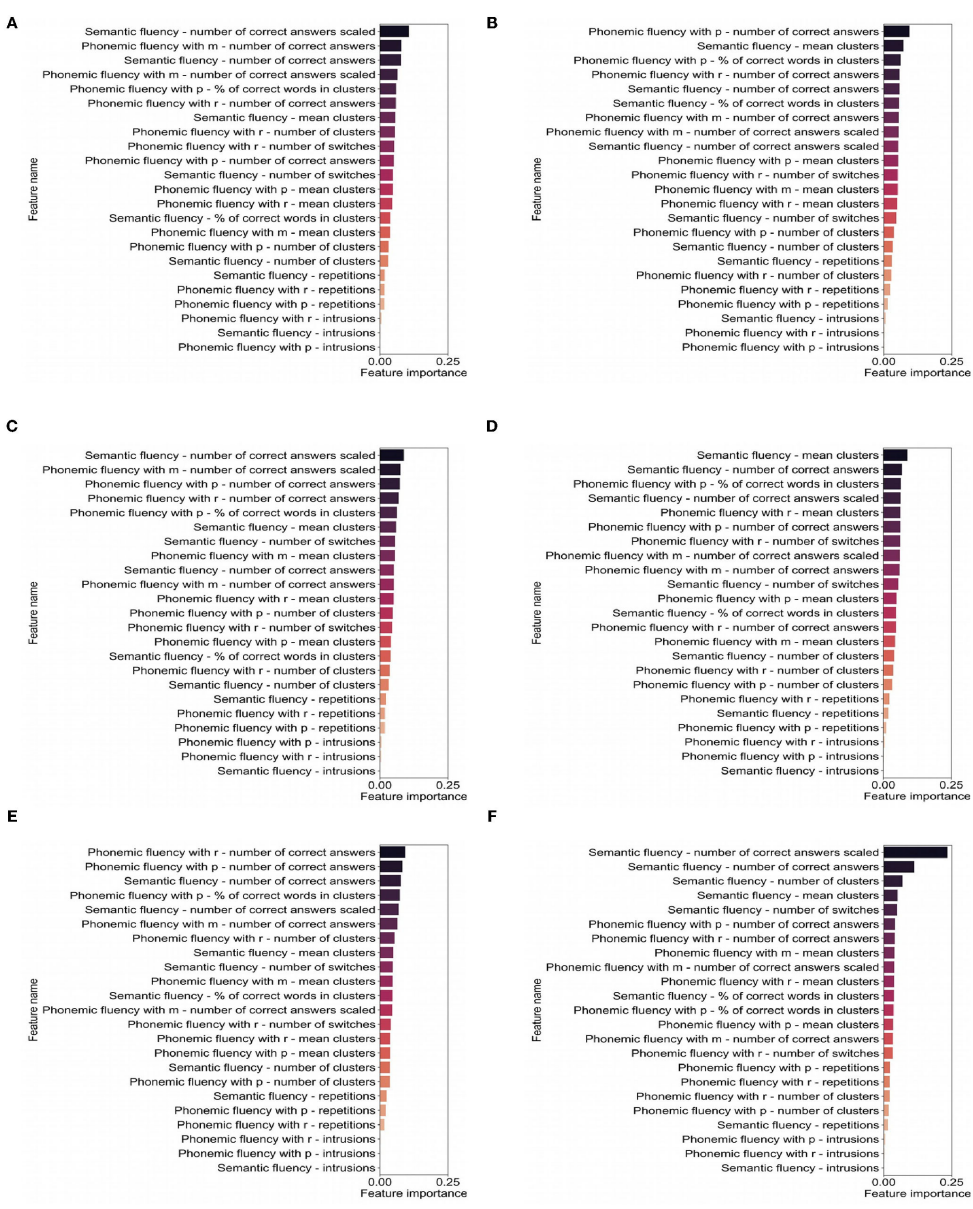
Features importances obtained with each Random Forest model for each classification task: **(A)** cognitive impairment, **(B)** attention and executive functioning, **(C)** information processing speed, **(D)** memory, **(E)** visuospatial function, and **(F)** language.

Two different classifiers (Support Vector Machine and Random Forest) were used to predict the presence of cognitive impairment, as well as the presence of cognitive dysfunction in each evaluated cognitive domain. Tuned hyperparameters and specifications of each model can be found in Supplementary Material 2. Figure 2 shows the F1-score obtained for each classifier, and full information about precision, recall, and F1-score values are depicted in Supplementary Material 3. Both aforementioned classifiers performed better for cognitive impairment and attention and executive dysfunction, with F1-scores between 67 and 71%. Conversely, classification performance scores for the other cognitive domains were lower. Features importances in Random Forest models are shown in Figure 3.

## Discussion

The cognitive processes involved in verbal fluency in MS remains controversial, due to the specific characteristics of cognitive impairment and brain damage associated with MS. In this study, we applied automatic linear modeling to investigate the neuropsychological tests that better explained the verbal fluency tests performance. Interestingly, we found different predictors according to the different fluencies (phonemic or semantic) and the different scores used (total words, clustering, and switching). These results support the view that fluency tasks provide useful information about a wide range of cognitive functions. Specifically, semantic fluency (total score) was predicted by the FCSRT (total free recall), Boston Naming Test, Stroop A, and PASAT, which confirm the influence of memory and language tasks, but also attention and time-dependent tests. Similarly, clustering in semantic fluency was predicted by the FCSRT, Stroop A, and Corsi test. Conversely, switching in semantic fluency was mainly explained by three attention-executive and time-dependent tests: Stroop A, ToL, and PASAT.

Regarding phonemic fluency, several tests measuring attention-executive functioning, language, and memory were the main predictors. Clustering was predicted by ToL and FCSRT, while switching by BNT, Stroop A, Corsi, ToL, FCSRT, and TMT-B. Thus, our results confirm the influence of three main cognitive domains in fluency tasks, including attention-executive functioning, memory, and language. Although the tests mainly associated with these cognitive domains are predictors of the different fluencies and scores, the importance of memory was greater in semantic fluency and clustering, and executive functioning in phonemic fluency and switching. In addition, it is worth mentioning that several of the best predictors were time-dependent tasks, which also emphasize a potential role of processing speed. Although the SDMT was not included in any statistical model, it showed moderate correlations with all the fluency scores, as in previous studies (6, 9). Overall, these findings emphasize the interest to extract several parameters in fluency tasks to capture as much information as possible.

Another interesting result is the role of the Boston Naming Test, which predicted several fluency scores, such as correct answers in semantic and phonemic fluency. This test shares some cognitive processes with fluency tasks, such as search, selection, and word retrieval, but with a lower degree of time restriction. Although language was usually considered to be largely preserved in MS, recent studies using novel tests evaluating the speed to lexical access have shown frequent impairment even in early stages (24).

We have developed several machine-learning algorithms trying to predict those patients with cognitive impairment, and those with dysfunction of specific cognitive domains. Interestingly, VF scores achieved acceptable values for the prediction of general cognitive impairment and executive dysfunction, which confirms the major role of executive functioning in VF in MS. Scores derived from phonemic fluency (e.g., correct words beginning with “p,” clusters, and switches) were more useful in the prediction of executive dysfunction. For general cognitive impairment prediction, a combination of scores from semantic and phonemic fluencies were amongst the most predictive, which suggests the interest of combining semantic and phonemic VF in short batteries (14). Unfortunately, the algorithms showed low levels of accuracy in the other cognitive domains, which supports the need for a full and comprehensive neuropsychological assessment to evaluate specific cognitive deficits in MS.

These findings may also be interpreted in terms of the neural basis of cognitive dysfunction in MS. Semantic fluency and phonemic fluency have been associated with subcortical volumes in voxel-based morphometry analysis (2). Specifically, phonemic fluency was mainly correlated with caudate, while semantic fluency with both thalamus and caudate in both hemispheres. Impairment of these structures is considered key in the pathophysiology of cognitive impairment in MS, especially in attention and executive functioning. Conversely, in other functions, such as memory or language, other regions are necessary to predict cognitive performance (i.e., hippocampus and temporal lobe in memory) (25). Neural basis of cognitive assessment in MS shows several particularities, in contrast with other disorders (tumors, stroke, or neurodegenerative dementias). In this regard, in other disorders VF has been mainly correlated with several cortical regions in the left hemisphere (26). These specificities warrant the study of the cognitive processes and neuroimaging correlates of the neuropsychological tests used in the setting of MS to accomplish an adequate interpretation of neuropsychological assessment.

Our study has some limitations. First, algorithms were developed on the basis of some criteria, which also included the impairment of VF. This could imply a certain degree of circularity in the machine learning analysis. However, these criteria were previously validated in an independent study, and impairment of VF according to these criteria was present in a relatively low percentage of cases classified as cognitively impaired (36.2% for semantic VF, and 29.8% for phonemic VF). Second, VF are tasks language-dependent, and our results should be confirmed in other cultures. In this regard, there are differences in the frequency of words between languages, and cross-cultural adaptations are required to minimize it, especially for phonemic fluency (27, 28). For instance, words beginning with “f,” “a,” and “s” are common phonemic fluency tasks for English speakers, but for Spanish speakers the initial letters “p,” “m,” and “r” have been proposed as an alternative and are generally preferred, based on the frequency of words (27–29). Third, we did not include neuroimaging analysis in this study. Correlation between the different scores and neuroimaging techniques (voxel-based morphometry, cortical thickness, diffusion tensor imaging, etc.) may be of interest in future studies. Fourth, we did not perform a correction considering motor dexterity. Due to the possibility of motor disorders in MS patients that could compromise the test interpretation, particularly in timed neuropsychological tests, this type of correction may be useful to improve the reliability of the neuropsychological examination (30). Finally, due to the aims of the study, a comprehensive battery was administrated with the possible presence of fatigue effect.

In conclusion, our study highlights the interest of further research into the assessment of VF in patients with MS. VF was influenced by many other cognitive processes, mainly including attention-executive functioning, episodic memory, and language. Semantic fluency and clustering were more explained by memory function, while phonemic fluency and switching were more related to executive functioning. The multiple cognitive components underlying VF tasks could serve for screening purposes. In this regard, we have developed several machine learning algorithms that could be useful to detect patients with cognitive impairment using only VF, although these models performed adequately only for general cognitive impairment and executive dysfunction. Overall, our study supports the implementation of a comprehensive and qualitative assessment of verbal fluency in MS, which may provide interesting insights into cognitive function in patients with MS.

## Data Availability Statement

The raw data supporting the conclusions of this article will be made available by the authors, without undue reservation.

## Ethics Statement

The studies involving human participants were reviewed and approved by Comite de Etica e Investigacion Clinical del Hospital Clinico San Carlos. The patients/participants provided their written informed consent to participate in this study.

## Author Contributions

AD-A, JM-G, and JAM-G: conceptualization and design of the study. AD-A, CD-A, AC-M, LV, PM-E, JM-G, and JAM-G: data curation. AD-A, LH-L, and JAM-G: formal analysis. JM-G: funding acquisitions. AD-A, CD-A, LH-L, AC-M, PM-E, VP, JM-G, and JAM-G: investigation. JM-G and JAM-G: supervision. AD-A and JAM-G: writing original draft. LH-L, CD-A, AC-M, LV, PM-E, VP, and JM-G: writing review and editing. All authors contributed to the article and approved the submitted version.

## Conflict of Interest

The authors declare that the research was conducted in the absence of any commercial or financial relationships that could be construed as a potential conflict of interest.
