# Supplementary material for: Cognitive Processes Underlying Verbal Fluency in Multiple Sclerosis
Source: Front Neurol. 2021 Jan 21;11:629183. doi: 10.3389/fneur.2020.629183 (PMC7859643; doi:10.3389/fneur.2020.629183)
Supplement: Supplementary file 1 [file Table_1.docx]

**Supplementary Material 1**

*Criteria for cognitive impairment in each domain according to the neuropsychological battery Neuronorma.*

| Impairment | Criteria |
| --- | --- |
| Attention and  executive functioning                  Information processing speed      Memory          Visuospatial function    Language | Impaired scores on TMT-B, but normal scores on A.  Impaired scores on total moves and correct total ToL scores.  Impaired Stroop C scores, but normal scores on A and B.  Raw verbal span forward score - backward score ≥ 3.  Raw Corsi's test forward score - backward score ≥ 3.  Impaired phonemic fluency, but normal semantic fluency.  Impaired phonemic and semantic fluency with normal scores on BNT.  Impaired SDMT scores.  Impaired TMT-A scores and another impairment on time-dependent tests (SDMT, Stroop, etc.)  Impaired FCSRT scores (trial 1 free recall, total free recall, total recall, delayed recall, delayed total recall) with normal scores on BNT.  ROCF-30min impaired scores, but normal scores on copy.  Impaired JLO scores.  Impaired ROCF scores (copy accuracy).  Impaired BNT scores.  Impaired semantic fluency, but normal scores on phonemic fluency. |
