# Supplementary material for: Cognitive Processes Underlying Verbal Fluency in Multiple Sclerosis
Source: Front Neurol. 2021 Jan 21;11:629183. doi: 10.3389/fneur.2020.629183 (PMC7859643; doi:10.3389/fneur.2020.629183)
Supplement: Supplementary file 2 [file Table_2.docx]

**Supplementary Material 2**

*Best models hyperparameters tuned with 5-Fold Cross-Validation Grid Search. SVM: Support Vector Machine*

| Class | Model | Hyperparameters |
| --- | --- | --- |
| Cognitive  impairment | SVM linear kernel  Random Forest | C = 100  n_estimators = 50, bootstrap = True,  max_features = ‘sqrt’ |
| Attention and executive functioning | SVM linear kernel  Random Forest | C = 0.01  n_estimators = 200, bootstrap = True,  max_features = ‘sqrt’ |
| Information processing speed | SVM linear kernel  Random Forest | C = 1000  n_estimators = 300, bootstrap = True,  max_features = ‘sqrt’ |
| Memory | SVM linear kernel  Random Forest | C = 100  n_estimators = 1000, bootstrap = True,  max_features = ‘sqrt’ |
| Visuospatial  function | SVM linear kernel  Random Forest | C = 10  n_estimators = 200, bootstrap = True,  max_features = ‘sqrt’ |
| Language | SVM linear kernel  Random Forest | C = 10  n_estimators = 200, bootstrap = True,  max_features = ‘sqrt’ |
