# Supplementary material for: Cognitive Processes Underlying Verbal Fluency in Multiple Sclerosis
Source: Front Neurol. 2021 Jan 21;11:629183. doi: 10.3389/fneur.2020.629183 (PMC7859643; doi:10.3389/fneur.2020.629183)
Supplement: Supplementary file 3 [file Table_3.docx]

**Supplementary Material 3**

*Precision, recall and F1-score values (%) obtained for each classification task (Class) with each model (SVM linear kernel and Random Forest). SVM: Support Vector Machine.*

| Class | Model | Precision | Recall | F1-Score |
| --- | --- | --- | --- | --- |
| Cognitive  impairment | SVM linear kernel  Random Forest | 63.64%  64.71% | 75.00%  78.57% | 68.85%  70.97% |
| Attention and executive functioning | SVM linear kernel  Random Forest | 50.85%  70.97% | 96.77%  70.97% | 66.67%  70.97% |
| Information processing speed | SVM linear kernel  Random Forest | 45.45%  52.00% | 55.56%  48.15% | 50.00%  50.00% |
| Memory | SVM linear kernel  Random Forest | 50.00%  50.00% | 34.78%  34.78% | 41.03%  41.03% |
| Visuospatial  function | SVM linear kernel  Random Forest | 50.00%  60.00% | 7.69%  23.08% | 13.33%  33.33% |
| Language | SVM linear kernel  Random Forest | 50.00%  50.00% | 44.44%  33.33% | 47.06%  40.00% |
